# A Rapid Assay to Detect Toxigenic *Penicillium* spp. Contamination in Wine and Musts

**DOI:** 10.3390/toxins8080235

**Published:** 2016-08-08

**Authors:** Simona Marianna Sanzani, Monica Marilena Miazzi, Valentina di Rienzo, Valentina Fanelli, Giuseppe Gambacorta, Maria Rosaria Taurino, Cinzia Montemurro

**Affiliations:** 1 Dipartimento di Scienze del Suolo, della Pianta e degli Alimenti, Università degli Studi di Bari Aldo Moro, Via G. Amendola 165/A, 70126 Bari, Italy; monicamarilena.miazzi@uniba.it (M.M.M.); valentina.dirienzo@gmail.com (V.d.R.); valentina.fanelli@uniba.it (V.F.); giuseppe.gambacorta@uniba.it (G.G.); cinzia.montemurro@uniba.it (C.M.); 2 Sinagri s.rl. Spin-off, Università degli Studi di Bari Aldo Moro, Via G. Amendola 165/A, 70126 Bari, Italy; 3 Agro.Biolab Laboratory s.r.l., SP240 km 13,800, 70018 Rutigliano (BA), Italy; taurinomariarosaria@gmail.com

**Keywords:** grape, *Penicillium* detection, mycotoxins, food safety

## Abstract

Wine and fermenting musts are grape products widely consumed worldwide. Since the presence of mycotoxin-producing fungi may greatly compromise their quality characteristics and safety, there is an increasing need for relatively rapid “user friendly” quantitative assays to detect fungal contamination both in grapes delivered to wineries and in final products. Although other fungi are most frequently involved in grape deterioration, secondary infections by *Penicillium* spp. are quite common, especially in cool areas with high humidity and in wines obtained by partially dried grapes. In this work, a single-tube nested real-time PCR approach—successfully applied to hazelnut and peanut allergen detection—was tested for the first time to trace *Penicillium* spp. in musts and wines. The method consisted of two sets of primers specifically designed to target the β-tubulin gene, to be simultaneously applied with the aim of lowering the detection limit of conventional real-time PCR. The assay was able to detect up to 1 fg of *Penicillium* DNA. As confirmation, patulin content of representative samples was determined. Most of analyzed wines/musts returned contaminated results at >50 ppb and a 76% accordance with molecular assay was observed. Although further large-scale trials are needed, these results encourage the use of the newly developed method in the pre-screening of fresh and processed grapes for the presence of *Penicillium* DNA before the evaluation of related toxins.

## 1. Introduction

Wine is one of the major processed grape (*Vitis vinifera* L.) products, with a worldwide production of 26,404,435 tons [1], obtained by the total or partial alcoholic fermentation of grapes or musts [2]. Usually, red wines are produced from black grape musts, and fermentation occurs in presence of the grape skins, whereas white wines are produced by fermentation of the juice obtained by pressing crushed grapes. The process stops either naturally, when sugars are completely converted, or artificially, by lowering the temperature. Musts can also undergo “enrichment”—that is, an increase in the sugar concentration prior to fermentation—to gain a proper final level of alcohol in the wine. However, fermenting musts are not only an intermediate product, as they are directly consumed in wine-growing areas of Northern Europe (mainly Germany and Austria) during the autumn season [3], in particular by children [4]. Their overall quality is usually poor, as they represent the wastes of the production of quality-tested wine. Therefore, the risk of contamination by toxic metabolites produced by grape-contaminating fungi (e.g., *Aspergillus* spp., *Penicillium* spp., *Alternaria* spp.) is relevant. Although *Aspergillus* and ochratoxin A are considered the main genus and mycotoxin associated to grapes, respectively [5], *Penicillium* is emerging as a cause of postharvest decay. For instance, Diaz et al. [6] collected 132 isolates—mainly *P. brevicompactum*, *P. expansum*, and *P. glabrum*—from apparently healthy grape clusters and in the air of vineyards and wineries, detecting the mycotoxin patulin in Cabernet Sauvignon musts, although its concentration decreased with fermentation. Therefore, mycotoxins may represent a serious concern, especially if contamination takes place after fermentation in environments dedicated to wine storage and bottling and in wines obtained from partially dried grapes. Indeed, Picco and Rodolfi [7] found high fungal counts in the bottling areas of industrial wineries, including *Penicillium* species such as *P. chrysogenum*, *P. citreonigrum*, *P. crustosum*, and *P. viridicatum*, whose constant presence potentially contaminate wines and may be hazardous to human health.

The 58 species reported in *Penicillium* subgenus *Penicillium* produce a large number of bioactive extrolites (secondary metabolites), including several mycotoxins (ochratoxins, citrinin, patulin, penicillic acid, verrucosidin, penitrem A, cyclopazonic acid, etc.) [8]. However, among them, only certain species and related metabolites are present on grapes. A major role is played by *P. expansum* and the toxin patulin [9], which is mutagenic, neurotoxic, immunotoxic, genotoxic, and has deleterious gastrointestinal effects in rodents [10]. Due to its toxicity, the World Health Organization (WHO) established a provisional maximum tolerable daily intake (PMTDI) of 0.4 μg/kg body weight [11]. Moreover, the European Commission established a maximum concentration of 50 μg/kg of patulin in fruit juices and nectars, reconstituted fruit juices, spirit drinks, cider, and other fermented drinks derived from or containing apples; 25 μg/kg for solid apple products; and 10 μg/kg for baby food [12]. Finally, other Countries outside Europe also set up regulatory limits—e.g., in Japan, the Ministry of Health, Labour, and Welfare (MHLW) adopted the maximum level of 50 μg/kg for apple juices [13]. In contrast, no regulation for patulin content in grapes and wines exists worldwide.

Some conventional PCR assays have been reported for the detection of *Penicillium* spp. [14,15,16]. However, the advent of real-time PCR (qPCR) permitted the more-efficient detection and quantification of *Penicillium* DNA in a wide variety of food matrices. For instance, a qPCR assay based on the β-tubulin gene was proposed to monitor *Penicillium* development on apples [17]. More recently, the innovative High Resolution Melting (HRM) technique was applied successfully to detect *Penicillium* spp. from apples, sweet cherries, and table grapes [9]. Finally, qPCR assays have been set up targeting patulin biosynthetic genes in terms of presence and expression [18,19]. However, most of the molecular assays targeting pathogens in biological matrices suffer from difficulties in extracting DNA of good quality and quantity, and from low pathogen representation.

The combined use of nested PCR and qPCR in a single-tube assay might enhance both the sensitivity and specificity of pathogen detection in foods. In fact, nested PCR allows the production of fragments with two different sizes, increasing the initial template. The use of the fluorescent molecule SYBR Green during the qPCR assay enables the direct monitoring of fragment production. Their combination in a single-tube could help to reduce time and cost of analysis, without losing efficiency. Bergerová et al. [20] and Costa et al. [21,22] used a similar approach for the detection of peanut, hazelnut, and almond allergens in food, respectively.

The aim of this work was to set up a diagnostic tool based on single-tube nested qPCR for the detection and semi-quantification of *Penicillium* spp. in musts and wines, as a quick and sensitive pre-screening of the putative presence of mycotoxins with health significance for consumers and economic significance for retailers.

## 2. Results

### 2.1. Set up of Experimental Design

**Figure 1:**
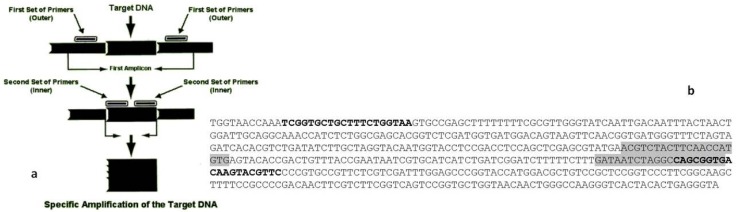
(**a**) Nested amplification scheme and (**b**) sequence of a portion of *Penicillium expansum* gene encoding β-tubulin (GenBank accession no. KC342829). “Outer” primers (NESF/NESR) in bold, and “inner” primers (HRMF/HRMR), shaded in grey, were designed on conserved portions.

Two sets of primer pairs designed upon a portion of β-tubulin gene, with different annealing temperatures, were used to detect the presence of *Penicillium* spp. in extracted samples. The first set of primers (NESF-NESR) generating PCR fragments of 320 bp worked as the “outer” primers to delineate the chosen target sequence (Figure 1). This primer pair was selected to hybridize at a higher temperature (60 °C), conferring selectivity to the reaction. The second set of primers, HRMF-HRMR, producing PCR fragments of 96 bp, was defined to act as “inner” primers at lower hybridization temperatures (55 °C). In order to perform the single-tube nested real-time PCR approach, two independent temperature phases were established. During phase 1, PCR fragments of 320 bp were amplified, to serve as DNA template later on in the reaction, with no fluorescence acquisition. Phase 2 was planned to obtain PCR fragments of 96 bp using the 320 bp fragments as template, and the collection of fluorescence was performed at the end of each cycle. The number of cycles to be used in each phase was selected according to the best performance in nested qPCR trials: phase 1 was set at 15 cycles, whereas phase 2—with fluorescence signal acquisition—was carried out using 30 cycles.

### 2.2. Specificity and Sensitivity Assay

In BLAST analyses, *Penicillium* primer sets did not match any of the available DNA sequences in international databases other than their reference genus. Moreover, specificity tests were conducted amplifying DNA from different fungal genera and species commonly associated to grape (Table 1). A positive amplification (increase of fluorescence) was obtained by the sole *Penicillium* strains. No cross-amplification with grape (*Vitis vinifera*) DNA was observed.

**Figure 2:**
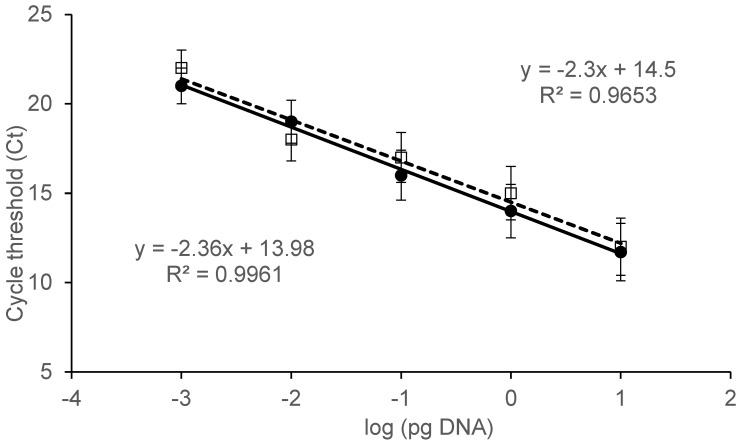
Linear relationship between *Penicillium* DNA concentration in the range 100–0.001 pg/μL and cycle threshold (Ct) given by the instrument. Standard curve, linear equation, and determination coefficient (*R*^2^) was determined by plotting Ct values against log (pg DNA) concentration (*x*-axis) in absence (●) and presence (□) of grape DNA. Error bars (indicating standard error of the mean, SEM) were obtained from three parallel experiments, in which each sample was run in triplicate.

To evaluate the sensitivity of the reaction and to quantify *Penicillium* DNA, a standard curve was drawn (Figure 2). Five 10-fold dilutions in the range 100–0.001 pg/μL of *P. expansum* DNA were amplified. The standard curve showed a linear correlation (*p* ≤ 0.001) between input DNA and Ct values, with *R*^2^ = 0.9961. The system was able to efficiently amplify up to 1 fg of target DNA.

In order to evaluate the influence of grape extracts on the quantification of fungal DNA, the experiment was repeated, adding grape DNA to all serial dilutions. The obtained curve was not influenced by the presence of grape DNA, since an identical detection limit and very similar determination coefficient (*R*^2^ = 0.9653) were observed (Figure 2).

### 2.3. Penicillium Detection in Real Samples

Eighty-two musts and wines (whites and reds) were collected from private wineries in Southern Italy. They came from tanks (large resin-coated cement underground containers), cisterns (large circular stainless steel vessels on legs), and silos (small cisterns). Samples underwent DNA extraction, and, in order to prevent false-negatives, their suitability to PCR amplification was confirmed using grape-specific primers. Of the analysed samples, 38 (46%)—made up of 19 musts (6 whites and 13 reds) and 19 wines (7 whites and 12 reds)—were found positive for *Penicillium* contamination (Table 2). In particular, they came from 18 (out of 31, 58%) tanks, 5 (out of 22, 23%) cisterns, and 15 (out of 28, 54%) silos. Therefore, there was a significantly lower frequency of contamination among samples coming from cisterns (Kruskal–Wallis, *p* < 0.05). *Penicillium* DNA was found in the range 0.001–2.634 pg/μL, with the red must SS26 and white wine T40 containing the higher and lower quantity of *Penicillium* DNA, respectively. However, there were no significant differences between musts/wines and reds/whites.

### 2.4. Patulin Quantification in Real Samples

Patulin occurrence and concentration was estimated for the seventeen samples of red and white musts and wines that resulted positive for the presence of *Penicillium* (Table 3). Thirteen of the analysed samples resulted contaminated in the range 27–1911 μg/L, with white wines T17 and SS21 as the most and least contaminated samples, respectively. There were no significant differences in terms of toxin contamination between musts and wines, or reds and whites. A concordance between presence/absence of the fungus and of the toxin was observed for 13 samples (76%), whereas in four samples, *Penicillium* but not patulin was detected. There was no linear correlation between *Penicillium* DNA and patulin contamination extents.

## 3. Discussion

*Penicillium* species are ubiquitous fungi associated with organic matter in nature. Although mainly linked to other commodities, their presence as epiphytes on grapes has been reported, with the frequency increasing considerably as berries mature [6]. However, species of *Penicillium* are gaining attention not only as grapevine pathogens at harvest [23], but also during the postharvest phase and winemaking [9,24].

Fungi cause drastic chemical and enzymatic modifications depending on grape variety and production stage [25,26], leading to serious sensory defects and risks of contamination by toxic metabolites (including patulin) in wine. Consequently, there is an increasing interest in determining contamination by *Penicillium* spp. of grapes, musts, and wines—especially those obtained from partially dried grapes. For example, the withering process for the production of passito wines (e.g., Amarone, Sfurzat, Vin Santo, Recioto) lasts up to 5 months in specific thermo-hygrometric conditions, in which fungal contamination can take place [27].

The correct evaluation of the potential presence of pathogens/metabolites using molecular assays is highly dependent on numerous factors, such as the type of food matrix, the mycotoxin/DNA markers, and the chosen methodology, among others [28]. In this work, we present an alternative method based on the assembly of two DNA-based techniques (nested PCR and real-time PCR) for the detection of *Penicillium* DNA in wines and musts. The task was to set up an assay that is easily applicable on a large number of samples at once, thus representing a quick and efficient pre-screening before traditional chemical analyses.

Regarding the nested real-time PCR assay developed in this work, our system was able to detect the presence of *Penicillium* in 46% of the tested samples, with samples coming from cisterns showing the lowest contamination. The detection limit was 1 fg, a result particularly interesting, considering that the average weight of the haploid genome of *Penicillium* spp. is reported to be 31 fg [29]. Moreover, this sensitivity level is much better than levels reported in literature concerning *Penicillium* detection in food matrices [17,18,19]. Indeed, by the introduction of the nested approach, it was possible to enhance the performance of a traditional real-time PCR assay. The proposed new detection system presents the advantage of high specificity conferred by the use of two pairs of primers at different annealing temperatures. In particular, the empirical rule for single-tube nested real-time PCR system—Ta (inner primers) < Ta (outer primers)—used for the detection of Ara h 3 [20], hsp1 [21], and Pru du 6 [22] allergens, was followed. The single-tube nested real-time PCR approach presented high performance criteria and apparent robustness, since it was not affected by shifts in temperature, time, cycle number (despite the existence of two different reaction protocols within the same assay), or the coexistence of grape DNA. Moreover, the single-tube amplification could be particularly efficient in preventing the cross-contamination and false negative results that are the major drawbacks of a nested approach.

As confirmation of *Penicillium* contamination, patulin presence was evaluated in representative samples. The mycotoxin was found in 71% of analysed wines and musts. With one exception (SS21), it was above the EU regulatory limit of 50 μg/kg foreseen for fermented apple juices, since there are no specific regulatory limits for patulin in wines and musts. The huge amount of toxin recorded even in wines strongly evidences the risks for consumers’ health, stating the need to detect and control the presence of patulin-producing fungi such as *Penicillium* all through the winemaking chain. The issue of the presence of patulin in grape musts was already addressed in Austria, as it was detected (maximum values 23.6–750 μg/kg) in 86 of the 164 samples surveyed from 1996 to 2000 [30]. This finding was alarming, considering that fresh grape must is offered to children as a non-fermented and unheated drink in the Austrian wine-growing regions [30].

A 76% accordance between molecular and toxicological data was recorded, although it was not quantitative. Similarly, Majerus et al. [3] found that contamination of grape must with patulin did not necessarily correlate with the moulding of the product, and Fredlund et al. [31] reported that the levels of both deoxynivalenol and zearalenone did not correlate with the DNA levels of *Fusarium culmorum* or other *Fusarium* species. In four samples containing *Penicillium* DNA, no patulin was detected. This was not surprising, since not all *Penicillium* species reported on grape are able to produce patulin [8]. Moreover, 60-plus species of moulds encompassing over 30 genera (including *Paecilomyces*, *Saccharomyces*, *Alternaria*, *Byssochlamys*, and *Aspergillus*) [32]—many of which have been reported on grape—produce patulin. In a recent study, the presence of patulin biosynthetic gene *patN* proved to be not predictive for patulin contamination [33]. Finally, it has to be considered that *Penicillium* produces several other toxic compounds (e.g., citrinin, chaetoglobosins, etc.) that can affect the quality of and safety of the product [34], and thus have to be monitored.

## 4. Conclusions

In conclusion, the single-tube nested real-time PCR method presented in this work constitutes an alternative, quick, and reliable approach for the detection of *Penicillium* even at trace levels in grape-derived products. The interesting results obtained with this approach highlight the usefulness of this new tool and its potential for the identification of pathogens in food matrices, for which further research work is needed. Moreover, the high patulin levels found in analyzed samples suggest the need to pay for greater attention to *Penicillium* toxins in musts and wines.

## 5. Materials and Methods

### 5.1. Sample Collection

During autumn 2013 and spring 2014, 82 musts and wines (whites and reds) were collected from private local wineries in the Apulia region, Southern Italy (Table 1). After 10 min of stirring, 6 L of each sample were collected, divided in three bottles of 2 L each, and stored at 4 °C until use. Among them, 17 samples were analyzed for patulin content.

### 5.2. DNA Extraction

DNA extraction from musts and wines was performed according to the method of di Rienzo et al. [35]. The DNA was further purified using the HiYield™ Gel/PCR Fragments Extraction Kit (Real Genomics, Banqiao City, Taiwan) according to manufacturer instructions, performing two washing steps and recovering the DNA with the elution buffer pre-heated at 60 °C. The DNA concentration, purity, and integrity were determined both by the Nano-Drop™ 2000 Spectrophotometer (Thermo Scientific, Waltham, MA, USA) and electrophoresis on a 0.8% agarose Tris/Borate/EDTA (TBE) gel. In order to prevent false negatives, the suitability of extracted DNA to PCR amplification was evaluated using *V. vinifera* primers [36].

### 5.3. Penicillium Detection System

Two sets of primers designed upon a portion of β-tubulin gene were used to detect the presence of *Penicillium* spp. in extracted samples. The inner primers HRMF/HRMR were those reported by Sanzani et al. [9], whereas the outer primers were NESF (5′-TCGGTGCTGCTTTCTGGTAA-3′) and NESR (5′-GAACGTACTTGTCACCGCTG-3′).

### 5.4. Nested One-Tube Real-Time PCR Assays

Real-time PCR assays were performed in 10 μL of total reaction volume. For each reaction tube, 5 μL of DNA, 1× SYBR^®^ Select Master Mix (Thermo Scientific), 300 nM of each inner primer HRMF1/HRMR1, and further 300 nM of each outer primer NESF/NESR were used. All real-time PCR assays were made on an iCycler iQ thermal cycler (BioRad, Hercules, CA, USA).

Nested real-time PCR assays were carried out with two different temperature programs: phase 1, performed without collecting fluorescence signal; and phase 2, with collection of the fluorescence signal at the end of each cycle. The number of cycles used in each phase was defined as follows: phase 1 from 5 to 15 cycles; phase 2 from 30 to 40 cycles. The following temperature protocol was used: 50 °C for 2 min, 95 °C for 2 min, 5–15 cycles at 95 °C for 15 s, 60 °C for 15 s (phase 1), and 30–40 cycles at 95 °C for 15 s, 55 °C for 15 s, and 72 °C for 15 s (phase 2). Fluorescence was acquired during the extension at 72 °C to further improve specificity and signal-to-noise ratio [37]. Data were collected and analyzed using the iCycler iQTM associated software (Real time Detection System Software, version 3.0, BioRad). Cycle threshold (Ct) values were calculated using the software at automatic threshold setting.

### 5.5. Specificity and Sensitivity Assay

To test the specificity of the reaction, the DNA of the most frequent fungal genera reported on grape, plus the DNA of grape and of *Penicillium* spp., was amplified as reported above.

Moreover, to assess the sensitivity of the assay, *Penicillium* DNA was serially diluted ten-fold with sterile water to yield final concentrations from 100 to 0.001 pg/μL, and amplified as described above. A standard curve was generated by plotting the DNA amounts [log (pg)] against the corresponding Ct value. Determination coefficient (*R*^2^) and linear equation were calculated. In order to evaluate the influence of co-extracted DNA on the efficiency of the two primer sets, a standard curve was drawn by adding 50 ng of *V. vinifera* DNA to each reaction mixture. *Penicillium* concentration in unknown samples was extrapolated from the standard curve.

### 5.6. Patulin Evaluation

For confirmation, the presence and concentration of patulin was evaluated in 17 samples positive to molecular assays.

#### 5.6.1. Chemicals and Reagents

All reagents had a purity >98.0% and were purchased from (Sigma Aldrich, Milan, Italy). A patulin stock solution in methanol was prepared at a concentration of 607.6 mg/L and stored at −20 °C. A working solution of 6.08 mg/L was also prepared.

#### 5.6.2. Extraction Procedure

An aliquot of 5 mL of wine/must was mixed with 5 mL of distilled water, 10 mL of acetonitrile, and 100 μL of internal standard (Dinoseb, (RS)-2,4-Dinitro-6-sec-butylphenol, 5 mg/L). The tube was shaken mechanically for 15 min. Then, a salt mixture (4 g MgSO_4_, 1 g NaCl, 1 g HOC(COONa)(CH_2_COONa)2·2H_2_O, and 0.5 g NaO_2_CCH_2_C(OH)(CO_2_H)CH_2_CO_2_Na·1·5H_2_O) was added to the tube, and a vigorous manual shaking was performed, followed by mechanical shaking for 15 min and centrifugation for 5 min at 3000× *g*. One mL of the raw extract was filtered on 0.22 μm regenerated cellulose filters (LLG Labware, Meckenheim, Germany) prior to liquid chromatography-tandem mass spectrometry (LC-MS/MS) analysis.

#### 5.6.3. Chromatographic and Mass Spectrometric Conditions

Analyses were performed by a Liquid Chromatograph NEXERA X2 LC30AD System (Shimadzu, Milan, Italy), equipped with a binary solvent delivery system, degasser, autosampler, and column heater. The separation was performed on a LUNA C8 analytical column (150 mm × 2 mm I.D.), with 5 μm particles, from Phenomenex (Torrance, CA, USA). The detection system was an AB SCIEX LC/MS/MS Triple Quad 5500 System tandem mass spectrometer equipped with an electrospray ionization interface (ESI) operating in the negative ion mode, using multiple reaction monitoring (MRM). A gradient elution was performed using a mobile phase (flow rate 0.25 mL/min) constituted by water (1% CH_3_COOH and 5 mM C_2_H_3_O_2_NH_4_) and methanol (1% CH_3_COOH and 5 mM C_2_H_3_O_2_NH_4_), eluent A and B, respectively. The program started at 10% eluent B and ramped to 40% at 5 min and to 90% at 11 min. It remained constant for 4 min and then decreased linearly to 10% of eluent B. This condition was kept constant for 5 min, and the column was re-equilibrated to the initial mobile phase composition. The column temperature was kept at 40 °C. The mass spectrometer ion source parameters applied were: Curtain Gas 30.00 psi; Desolvation Gas Temperature 550.00 °C; GS1 (air) 60.00 psi; GS2 (air) 55.00 psi; Ion Spray −4500.00 V. Collision energy and cone voltage acquisition parameters are reported in Table 4. The instrument had a limit of detection (LOD) of 0.02 mg/L and a limit of quantitation (LOQ) of 0.05 mg/L. The recovery of the method was in the range 81%–92%. Unknown samples were analyzed comparing standard patulin retention time and ion ratio (within ±20%); quantification was performed by a six-point calibration curve (*y* = 5479.74*x* + 554.75, *R*^2^ > 0.99) obtained for the mass fragment 152.9 → 108.9.

### 5.7. Statistical Analysis

Data processing and correlation analyses were performed using the statistical software package Statistics for Windows (StatSoft, Tulsa, OK, USA). Values were tested independently for normality using the Shapiro–Wilk (SW) test. Given that samples did not come from normally-distributed populations (SW test, *p* < 0.01), nonparametric tests were chosen for downstream analyses. The two-ways Wilcoxon (W) test and the Kruskal–Wallis (KW) test were applied to compare samples from two and three or more classes, respectively.

## Figures and Tables

**Table 1:** Results of nested real-time PCR amplifications of gene applied to fungal genera more frequently reported on grapes. *Penicillium* spp. and grape DNA were included as controls.

Isolate Code	Organism	Presence of Amplification Product
Pex6	*Penicillium expansum*	+
Pex29	*Penicillium chrysogenum*	+
Pex30	*Penicillium crustosum*	+
A64	*Alternaria alternata*	-
FV52	*Botrytis cinerea*	-
FV509	*Monilia laxa*	-
FV139	*Phellinus ignarius*	-
FV366	*Sclerotinia sclerotiorum*	-
FV155	*Cladosporium* spp.	-
FV406	*Aspergillus* spp.	-
FV150	*Rhizopus stolonifer*	-
FV126	*Fusarium* spp.	-
VV1	*Vitis vinifera*	-

**Table 2:** Samples used in the experiments, with type, storage modality, and nested real-time PCR results for the detection of *Penicillium* DNA.

Sample Code	Type	Storage	DNA Concentration (pg/μL)
C10	White must	Cistern	-
C11a	White wine	Cistern	1.007
C11b	White must	Cistern	-
C12a	Red must	Cistern	0.010
C12b	White must	Cistern	-
C13	White must	Cistern	-
C14	White must	Cistern	-
C15	White must	Cistern	-
C19	White must	Cistern	-
C21	White must	Cistern	-
C22	White must	Cistern	-
C23	White must	Cistern	-
C35	White wine	Cistern	-
C43	White must	Cistern	-
C47	White must	Cistern	-
C48	White must	Cistern	-
C52	White must	Cistern	-
C53	Red must	Cistern	-
C55	Red must	Cistern	0.016
C56	Red must	Cistern	-
C57	Red must	Cistern	0.003
C59	Red must	Cistern	0.006
SS5	White must	Silos	0.011
SS8	Red must	Silos	0.029
SS10	Red wine	Silos	1.0074
SS13	Red wine	Silos	-
SS14	Red must	Silos	0.010
SS15	White must	Silos	0.002
SS17	Red wine	Silos	0.002
SS19	Red wine	Silos	-
SS21	White wine	Silos	0.002
SS25	Red must	Silos	1.96
SS26	Red must	Silos	2.634
SS27	Red must	Silos	-
SS28	Red must	Silos	-
SS29	Red must	Silos	0.014
SS33	White must	Silos	0.003
SS34	White must	Silos	0.010
SS36	White wine	Silos	-
SS39	White must	Silos	0.056
SS42	Red must	Silos	-
SS44	Red wine	Silos	-
SS45	White wine	Silos	0.002
SS47	Red wine	Silos	-
SS48	Red wine	Silos	-
SS51	Red must	Silos	0.056
SS52	Red must	Silos	-
SS73	White wine	Silos	-
SS75	Red wine	Silos	-
SS77	Red wine	Silos	-
T2	Red wine	Tank	0.034
T7	Red must	Tank	0.183
T11	Red must	Tank	-
T13	Red must	Tank	0.034
T15	Red must	Tank	-
T17	White wine	Tank	0.006
T20	Red wine	Tank	-
T21	Red wine	Tank	0.011
T23	Red wine	Tank	0.010
T24	White wine	Tank	-
T25	Red wine	Tank	-
T26	Red wine	Tank	-
T27	Red wine	Tank	-
T28	Red wine	Tank	0.065
T31	Red wine	Tank	0.070
T32	White wine	Tank	0.042
T33	Red wine	Tank	0.010
T35	Red wine	Tank	0.309
T36	Red wine	Tank	-
T38	Red wine	Tank	0.029
T40	White wine	Tank	0.001
T41	White wine	Tank	0.023
T44	Red wine	Tank	-
T45	Red wine	Tank	-
T48	White wine	Tank	0.014
T49	Red wine	Tank	-
T51	Red wine	Tank	-
T52	Red must	Tank	-
T55	Red must	Tank	0.016
T58	Red wine	Tank	0.010
T70	Red wine	Tank	0.070

**Table 3:** Liquid chromatography-tandem mass spectrometry (LC-MS/MS) analysis for presence of patulin in musts and wines samples resulted positive to *Penicillium* DNA.

Sample Code	Type	*Penicillium* DNA Concentration (pg/μL)	Patulin Concentration (μg/L)
SS8	Red must	0.029	0
SS10	Red wine	1.0074	173
SS14	Red must	0.010	277
SS17	Red wine	0.002	397
SS21	White wine	0.002	27
SS29	Red must	0.014	154
SS33	White must	0.003	778
SS34	White must	0.010	0
SS39	White must	0.056	65
T13	Red must	0.034	60
T17	White wine	0.006	1911
T21	Red wine	0.011	712
T35	Red wine	0.309	82
T40	White wine	0.001	681
T41	White wine	0.023	0
T48	White wine	0.014	0
T58	Red wine	0.010	669

**Table 4:** Optimization of the collision energy and cone voltage for patulin by infusion of the mycotoxin directly into the LC effluent, and final acquisition parameters.

ID	Q1 Mass (Da)	Q3 Mass (Da)	Dwell (msec)	DP	EP	CE	CXP
Patulin 1	152.9	109.0	5.00	−45.00	−10.00	−13.00	−7.00
Patulin 2	152.9	80.9	5.00	−45.00	−10.00	−15.00	−7.00
Dinoseb 1	239.0	133.9	5.00	−120.00	−8.00	−58.68	−10.00
Dinoseb 2	239.0	163.1	5.00	−120.00	−8.00	−41.78	−10.00

DP: declustering potential; EP: entrance potential; CE: collision energy; CXP: cell exit potential.
